# Minority identity and social structures shape diffusion dynamics of minority languages: a combined macro and micro approach

**DOI:** 10.1098/rsos.250011

**Published:** 2025-04-16

**Authors:** Ya Gao, Wenqi Liu

**Affiliations:** ^1^ Faculty of Science, Kunming University of Science and Technology, Kunming, People’s Republic of China

**Keywords:** language diffusion, minority identity, phase transition, complex network, real social network

## Abstract

Language is a tool for cultural communication, and diffusion is influenced by many factors. However, many studies have highlighted the importance of language status, while the critical factor of minority identity has been neglected. Minority identity is a sociological factor reflecting individual preferences for minority languages. Here, we introduce a framework for characterizing the language diffusion within minority groups, leading to the emergence of new ethnolinguistic phenomena: language segregation and coexistence. This finding challenges the previous assumption that language status alone determines language dynamics. Furthermore, we add a self-minority identity transmission mechanism to understand how language diffusion occurs. Monte Carlo simulations and theoretical analyses reveal that self-minority identity transmission significantly fosters minority language diffusion in both heterogeneous and homogeneous networks, especially in heterogeneous networks, and that increasing the average degree of the network promotes minority language diffusion. Finally, we apply a real-world social network in the Wa minority region of Yunnan, China, to validate that minority language diffusion exhibits a phase transition and that the critical threshold depends on the network structure and the diffusion of self-minority identity. Moreover, we deepen the theoretical understanding of sociolinguistics and provide a theoretical basis and policy recommendations for protecting and promoting minority languages.

## 1. Introduction

Language is a complex, multilayered social phenomenon that emerges through human interactions and evolves over time. The evolution of language reflects changes in human societies and cultures, providing insights into the dynamics of communication within and across social groups [1]. The global diversity of languages and their uneven distribution highlight the complex interplay of historical, cultural and social factors shaping language evolution [2,3]. These factors drive language shifts, the emergence of new languages and even the extinction of some languages. Much like global biodiversity, the world’s linguistic diversity is under threat, with over 6000 languages estimated worldwide, half of which are predicted to face extinction within this century [4]. The loss of linguistic diversity undermines human cultural richness by eroding unique knowledge systems, traditional practices and diverse worldviews, particularly those essential for addressing global challenges like climate change and biodiversity conservation. Additionally, language loss disrupts cultural identity and intergenerational continuity, diminishing humanity’s collective cultural heritage and weakening the social fabric of affected communities.

To protect the diversity of languages and make them coexist, the development of human society should be traced. A pioneering study [5] introduced autonomous differential equations to the study of language competition for the first time. Daniel *et al.* [6] further have used differential equation dynamics model (A-S model) to describe the competition between two languages, predicted language extinction due to low language status and finally validated the model with real data. The A-S model opens the door to the study of language competition using dynamical equations. Subsequent articles have provided insights into how languages evolve and compete under the influence of different factors through mathematical modelling [7–15]. By analysing the similarity of the two languages [7], a similarity coefficient between the languages is proposed and it is concluded that as long as the two languages are similar enough, the languages will not die out. Based on the A-S model, the horizontal and vertical transmission of language has been proposed to better restore the real transmission path of language [8]. Language interactions between populations also have an impact on language transmission [9], where protective measures for endangered languages are also taken into account, which increases the likelihood of language coexistence. The influence of geographic factors [10] was taken into account in the study to extend the A-S model to spatial factors. Intraregional complexity has a significant effect on language migration, and language migration rates are inversely proportional to intraregional complexity [11]. To better explain the phenomenon of speech transmission, a model of social interaction based on surface tension was proposed to simulate the geographic distribution of real dialect boundaries [12], that the main influences on language decline are mainly geographic and demographic distributions. By incorporating spatial factors [13], it is shown that the most important determinant of the spread and retreat of a language is the interaction among individuals who speak the same language. However, most studies on language competition lack evidence of actual data, a deficiency for which Gong *et al*. [14] proposed a parameter estimation method. Recently, ecological factors of global language diversity have been analysed, revealing that language diversity is influenced by climatic factors [15]. However, the above model ignores the influence of sociological minority identity factors on language diffusion.

Minority identity is contained in attitudes, and attitudes have a significant impact on language evolution [16–18]. In this context, the influence of two attitudinal dimensions on language evolution, language status and language solidarity has been revealed through a global cross-cultural study [19,20]. Attitudes can be categorized as positive or negative. Positive attitudes favour certain languages, while negative attitudes drive people to practice prejudice and discrimination when they use a language for communication and thus not to use it [21,22]. Rocha *et al.* [23] considered the factor of ideology, where the process of spreading an ideology is similar to the SIR model of epidemics [24–26], where S denotes a non-enthusiastic individual who belongs to a certain cultural organization and is in contact with the enthusiastic individual of a certain culture becoming one of them after contacting I, who belongs to an active spreader, and R denotes the change from an enthusiastic individual I to a non-enthusiastic individual R with the passage of time. Other applications of the SIR model are to thought and culture [27–29]. However, in minority areas, the distribution of minorities is in the form of aggregation, and the competition is between different minority languages, which have roughly the same language status and number of people. For example, in Yunnan Province, the vast majority of minority groups are distributed in focused formations, such as the minority Lahu and the minority Wa, which are distributed in the southwestern part of Yunnan Province and live in the same mountainous area; the minority Nu and minority Bai, who live in Dali Prefecture and live in the same river basin area. What are the effects of different minority identities of different ethnic groups on language diffusion in the areas minorities are concentrated?

To investigate the impact of minority identity on the competition between different minority languages within the same region, we propose a framework to characterize the group dynamics of language diffusion under the influence of minority identity. This framework consists of five states in which minority identity is considered part of an individual’s attitudinal structure, both cognitive and behavioural [30–32]. In this study, an individual’s minority identity is reflected through their language choice behaviour: when minority identity is strong, individuals tend to choose and persist in using their own minority language; conversely, when identity is weak, individuals are more likely to choose other languages. Moreover, complex networks play a crucial role in language transmission dynamics [33–35]. We analyse the effect of minority identity on language evolution in complex networks. To simplify the system, we model it as a binary interaction framework, where ‘binary interaction’ refers to two individuals in the network who interact directly. Within this framework, each individual possesses varying levels of minority identity, and their state is continuously updated through direct interactions with their neighbours. These interactions follow a set of predefined behavioural rules, which represent simplified assumptions about language choice and diffusion. Specifically, individuals decide which language to choose during interactions based on the strength of their minority identity. This sense of identity can be either reinforced or weakened through network structures and interactions, thereby influencing individuals' language choices. Our model not only considers the actual language usage states of individuals but also introduces minority identity as an internal preference mechanism, revealing its role in the process of language diffusion. The study shows that under the influence of varying levels of minority identity, different linguistic phenomena may emerge, including language extinction, language isolation and language coexistence. Through this framework, we systematically explore how the interaction between minority identity and network structure shapes the overall dynamics of language evolution. This research deepens our understanding of language competition mechanisms and provides a theoretical foundation and policy recommendations for the protection and revitalization of minority languages.

This article is organized as follows. The language diffusion model with minority identity is introduced in §2. Section 3 introduces the basic properties of the model, including some new language phenomena. Section 4 presents the influence of self-minority identity transmission in complex network. Section 5 presents the validation of real minority social networks. Section 6 presents the conclusion.

## 2. Model

**Figure 1.**
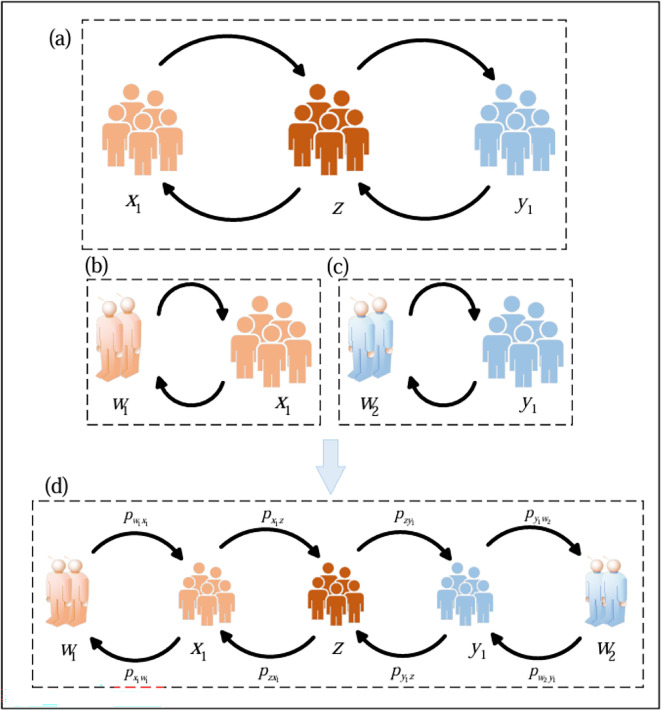
The schematic diagram of language diffusion with minority identity. This society consists of many speakers who speak evenly with other individuals. The speakers are categorized into five states, $w_{1}$ is only speaking own minority language A, $x_{1}$ can speak own minority language A and can learn other minority language B, $w_{2}$ is only speaking own minority language B, $y_{1}$ can speak own minority language B and can learn other minority language A, bilinguals z speaking minority language A and minority language B. Language transmission is (a), (b) and minority identity transmission (c)**.** The overall transmission process is (d). The transmission process consists of language transmission and minority identity transmission.

To research the influence of minority identity on language diffusion, we consider a five-state model in which the population can be divided into five groups X, Y, Z and $W_{1}$, $W_{2}$. Another aspect of minority identity is the degree of interest or openness towards foreign minorities. A strong sense of minority identity may sometimes manifest as resistance or rejection of foreign cultures, which is often reflected in a preference for speaking only their own minority language. A weak sense of minority identity may sometimes manifest as the acceptance of foreign cultures, which is often reflected in the ability to speak other languages. The population includes three types of speakers, the proportion of bilingual speakers (*z*), individuals who speak both languages A and languages B. The proportion of speakers who accept a minority B culture ($x_{1}$): Individuals who speak primarily language A but are supportive of foreign cultures and can also learn another minority language B. The proportion of speakers who accept a minority A culture ($y_{1}$): Individuals who speak primarily language B but are supportive of foreign cultures and can also learn another minority language A. The proportion of speakers who resist a minority B culture ($w_{1}$): Individuals who oppose foreign cultures and exclusively speak their own minority language A. The proportion of speakers who resist a minority A culture ($w_{2}$): Individuals who oppose foreign cultures and exclusively speak their own minority language B. ${x_{1}+y_{1}+z+w}_{1}+w_{2}=1$. Figure 1 shows these five groups, as well as the rates of transitions between the groups and the possible transitions.

Language state transmission can be categorized into two types: vertical transmission and horizontal transmission. Vertical transmission indicates that with probability μ (mortality), adults are replaced by children, language transmission follows that if parents are speakers with cultural resistance, then children also adopt this state, speaking only their own minority language. Children of bilingual parents choose one of the languages A, where the language B is their mother tongue, $x_{1}\to x_{1}$, $y_{1}\to y_{1}$, z→z. With respect to minority identity, where children’s minority identity originates from their parents, $w_{1}\to w_{1}$, $w_{2}\to w_{2}$. In horizontal transmission, bilingual adults can choose to switch between states or languages through interaction, expressed as $z\to x_{1}$, $z\to y_{1}$, z→z. However, in the process of horizontal transmission of minority identity, $x_{1}$ becomes $w_{1}$, $y_{1}$ becomes $w_{2}$ and the opposite can be established, $x_{1}\to w_{1}$, $y_{1}\to w_{2}$, $w_{1}\to x_{1}$, $w_{2}\to y_{1}$.

The language transmission rate depends on both the status of the languages A and *B* and their interaction values. The status of language *A* is indicated by $s_{1}$, and the status of language *B* is indicated by $s_{2}$, with $s_{1}+s_{2}=1.$ Interaction rate for state i to j is denoted by $I_{ij}$. The transmission rates $p_{zx_{1}}$ and $p_{zy_{1}}$ describe the transition from the state z to the states $x_{1}$ and $y_{1}$, respectively. Specifically, $p_{zx_{1}}$ is proportional to the status of language *A* ($s_{1}$), and $p_{zy_{1}}$ is proportional to the status of language *B* ($s_{2}$)；a larger $s_{1}$ or $s_{2}$ increases the respective transmission rates. In the reverse direction, the transition probability from $x_{1}$ or $y_{1}$ to z depends on the status of the attracting language, interaction rate and the size of the bilingual group. Similar to [36], the effect of the size of the bilingual group is the importance of the bilingual group as language *A* and language *B* for groups X and groups Y, expressed as $x_{1}+\beta z$ and $y_{1}+\alpha z$，where $\alpha ,\beta \in \left(0,1\right)$. Additionally, the transition of minority identity occurs through transitions between the two identity states. The rate of transition from $x_{1}$ to $w_{1}$ is proportional to the strength of minority identity ${1-c}_{1}$, the number of groups and the rate of interaction. Similarly, the diffusion rate from $y_{1}$ to $w_{2}$ is proportional to ${1-c}_{2}$, the group size and the interaction rate. Notably, the smaller the parameters $c_{1}$ or $c_{2}$, the stronger the minority identity, resulting in higher diffusion rates between identity states. The specific state transmission rate is shown in equation (2.2). Table 1 presents a brief explanation of the variables and parameters used in the language transmission model.

**Table 1.** Brief detail of parameters of language diffusion model with minority identity.

parameters	detail	parameters	detail
$x_{1}$	the proportion of speakers who accept a minority B culture	$s_{2}$	the status of languageB
$y_{1}$	the proportion of speakers who accept a minority A culture	$I_{ij}$	interaction rate for state i to j
z	the proportion of bilingual speakers	α	the importance of bilingual groups as language A
$w_{1}$	the proportion of speakers who resist a minority B culture	β	the importance of bilingual groups as language B
$w_{2}$	the proportion of speakers who resist a minority A culture	${1-c}_{1}$	the strength of minority identity A
μ	mortality	${1-c}_{2}$	the strength of minority B identity
$s_{1}$	the status of language A		

Based on the assumption that shift in the proportion of each group in the total population is given by the following system of nonlinear ordinary differential equations (mean-field approach) is as follows:


(2.1) $$\left\{ \begin{array}{l} \frac{dx_{1}}{dt}=w_{1}p_{w_{1}x_{1}}+zp_{zx_{1}}-x_{1}\left[\;p_{x_{1}z}+\;p_{x_{1}w_{1}}\right],\\ \frac{dy_{1}}{dt}=w_{2}p_{w_{2}y_{1}}+zp_{zy_{1}}-y_{1}\left[\;p_{y_{1}z}+\;p_{y_{1}w_{2}}\right],\\ \frac{dw_{1}}{dt}=x_{1}p_{x_{1}w_{1}}-w_{1}p_{w_{1}x_{1}},\;\;\;\;\;\;\;\;\;\;\;\;\;\;\;\;\;\;\;\;\;\;\;\;\;\;\;\;\;\;\;\;\\ \frac{dw_{2}}{dt}=y_{1}p_{y_{1}w_{2}}-w_{2}p_{w_{2}y_{1}},\;\;\;\;\;\;\;\;\;\;\;\;\;\;\;\;\;\;\;\;\;\;\;\;\;\;\;\;\;\; \end{array}\right.\;\;\;\;\;\;\;\;\;\;\;\;\;\;\;\;\;\;\;\;\;\;\;\;\;\;\;\;\;\;\;\;\;\;\;\;\;\;\;\;\;$$

where the transition rates are accordingly proportional to the total number of speakers, language status, the interaction value and minority identity of the language:


(2.2) $$\begin{array}{l} p_{x_{1}z}=\left(1-\mu \right)I_{x_{1}z}\left(1-s_{1}\right)\left(y_{1}+\alpha z\right),p_{y_{1}z}=\left(1-\mu \right)I_{y_{1}z}s_{1}\left(x_{1}+\beta z\right),\;\;\;\;\;\;\;\;\;\;\;\;\;\;\;\;\;\;\;\;\;\;\;\;\;\;\;\;\;\;\;\;\;\;\\ p_{zx_{1}}=\mu I_{zx_{1}}s_{1}x_{1},p_{zy_{1}}=\mu I_{zy_{1}}\left(1-s_{1}\right)y_{1},p_{w_{1}x_{1}}=\left(1-\mu \right)I_{w_{1}x_{1}}\left(1-c_{1}\right)x_{1},\;\;\;\;\;\;\;\;\;\;\;\;\;\;\;\;\;\;\\ p_{w_{2}y_{1}}=\left(1-\mu \right)\;I_{w_{2}y_{1}}c_{2}y_{1},p_{x_{1w_{1}}}=\left(1-\mu \right)I_{x_{1}w_{1}}c_{1}w_{1},p_{y_{1}w_{2}}=\left(1-\mu \right)I_{y_{1}w_{2}}c_{2}w_{2}.\;\;\;\;\;\;\;\;\;\;\; \end{array}$$

In the remainder of this article, we will focus on the case where vertical and horizontal transmission are equally likely, μ=0.5, $0.5\cdot I_{zx_{1}}=0.5\cdot I_{zy_{1}}=0.5\cdot I_{y_{1}z}=0.5$$\cdot I_{x_{1}z}=0.5\cdot I_{x_{1}w_{1}}=0.5$$\cdot I_{y_{1}w_{2}}=0.5\cdot I_{w_{1}x_{1}}=0.5$$\cdot I_{w_{2}y_{1}}=0.5\cdot I_{1}$. Let a time rescaling take place in the system in which the time interval dt is replaced by another time interval dt where $dt={\left(0.5I_{1}\right)}^{-1}dt$. Thus, the model is simplified to


(2.3) $$\left\{ \begin{array}{l} \frac{dx_{1}}{dt}=x_{1}\left[\left(s_{1}-\alpha \left(1-s_{1}\right)\right)\left(1-x_{1}-y_{1}-w_{1}-w_{2}\right)-\left(1-s_{1}\right)y_{1}-\left(1-2c_{1}\right)w_{1}\right].\;\;\;\;\;\\ \frac{dy_{1}}{dt}=y_{1}\left[\left(\left(1-s_{1}\right)-\beta s_{1}\right)\left(1-x_{1}-y_{1}-w_{1}-w_{2}\right)-s_{1}x_{1}-\left(1-2c_{2}\right)w_{2}\right].\;\;\;\;\;\;\;\;\;\;\;\;\;\;\;\;\;\\ \frac{dw_{1}}{dt}=x_{1}w_{1}\left(1-2c_{1}\right).\;\;\;\;\;\;\;\;\;\;\;\;\;\;\;\;\;\;\;\;\;\;\;\;\;\;\;\;\;\;\;\;\;\;\;\;\;\;\;\;\;\;\;\;\;\;\;\;\;\;\;\;\;\;\;\;\;\;\;\;\;\;\;\;\;\;\;\;\;\;\;\;\;\;\;\;\;\;\;\;\;\;\;\;\;\;\;\;\;\;\;\;\;\;\;\;\;\;\;\;\;\;\;\;\;\;\;\;\;\;\;\;\;\;\;\;\;\;\\ \frac{dw_{2}}{dt}=y_{1}w_{2}\left(1-2c_{2}\right).\;\;\;\;\;\;\;\;\;\;\;\;\;\;\;\;\;\;\;\;\;\;\;\;\;\;\;\;\;\;\;\;\;\;\;\;\;\;\;\;\;\;\;\;\;\;\;\;\;\;\;\;\;\;\;\;\;\;\;\;\;\;\;\;\;\;\;\;\;\;\;\;\;\;\;\;\;\;\;\;\;\;\;\;\;\;\;\;\;\;\;\;\;\;\;\;\;\;\;\;\;\;\;\;\;\;\;\;\;\;\;\;\;\;\;\;\; \end{array}\right.\;\;$$

### 2.1. Existence of equilibria

To find equilibria points, let the right-hand side of equation (2.3) be zero to obtain equation (2.4):


(2.4) $$\left\{ \begin{array}{l} x_{1}\left[\left(s_{1}-\alpha \left(1-s_{1}\right)\right)\left(1-x_{1}-y_{1}-w_{1}-w_{2}\right)-\left(1-s_{1}\right)y_{1}-\left(1-2c_{1}\right)w_{1}\right]=0.\;\;\;\;\;\;\;\\ y_{1}\left[\left(\left(1-s_{1}\right)-\beta s_{1}\right)\left(1-x_{1}-y-w_{1}-w_{2}\right)-s_{1}x_{1}-\left(1-2c_{2}\right)w_{2}\right]=0.\;\;\;\;\;\;\;\;\;\;\;\;\;\;\;\;\;\;\;\;\\ x_{1}w_{1}\left(1-2c_{1}\right)=0.\;\;\;\;\;\;\;\;\;\;\;\;\;\;\;\;\;\;\;\;\;\;\;\;\;\;\;\;\;\;\;\;\;\;\;\;\;\;\;\;\;\;\;\;\;\;\;\;\;\;\;\;\;\;\;\;\;\;\;\;\;\;\;\;\;\;\;\;\;\;\;\;\;\;\;\;\;\;\;\;\;\;\;\;\;\;\;\;\;\;\;\;\;\;\;\;\;\;\;\;\;\;\;\;\;\;\;\;\;\;\;\;\;\;\;\;\;\;\;\;\\ y_{1}w_{2}\left(1-2c_{2}\right)=0.\;\;\;\;\;\;\;\;\;\;\;\;\;\;\;\;\;\;\;\;\;\;\;\;\;\;\;\;\;\;\;\;\;\;\;\;\;\;\;\;\;\;\;\;\;\;\;\;\;\;\;\;\;\;\;\;\;\;\;\;\;\;\;\;\;\;\;\;\;\;\;\;\;\;\;\;\;\;\;\;\;\;\;\;\;\;\;\;\;\;\;\;\;\;\;\;\;\;\;\;\;\;\;\;\;\;\;\;\;\;\;\;\;\;\;\;\;\;\; \end{array}\right.\;\;\;\;\;\;\;\;\;\;\;$$

We can detect $E_{0}\left(0,0,0,0\right),E_{1}\left(1,0,0,0\right),E_{2}\left(0,1,0,0\right),E_{3}\left(0,0,1,0\right),E_{4}\left(0,0,0,1\right)$ by a simple check of model (3). I will refer to them as fixed points $E_{5}\left(2c_{2}-1/S_{1}+2c_{2}-1,0,0,S_{1}/S_{1}+2c_{2}-1\right)$, $E_{6}\left(0,1-2c_{1}/S_{1}-2c_{1},S_{1}-1/S_{1}-2c_{1},0\right)$, $E_{7}(\alpha s_{1}^{2}-\alpha s_{1}-2s_{1}+s_{1}^{2}+1/$$\alpha s_{1}^{2}-\alpha s_{1}-\beta s_{1}-s_{1}+\beta s_{1}^{2}+s_{1}^{2}+1,$$\beta s_{1}^{2}-\beta s_{1}s_{1}^{2}/$$\alpha s_{1}^{2}-\alpha s_{1}-\beta s_{1}-s_{1}+\beta s_{1}^{2}+s_{1}^{2}+1,0,0)$.

To better understand and illustrate the linguistic phenomenon of equilibrium points, we discuss the phenomenon of equilibrium points appearing as described above and the reasons explaining the linguistic phenomenon appearing.

## 3. Basic properties of language diffusion model

*Proposition 1. Language complete extinction*

*Case 1*: When $s_{1}>1/2$, high status role for language A and weak minority identity, the phenomenon of language complete extinction is formed, both in the sense that only individuals $x_{1}$ remain, specifically denoted as **E1**.

*Case 2*: When$\;s_{1}<1/2$, high status role for language B and weak minority identity, the phenomenon of language complete extinction is formed, both in the sense that only individuals $y_{1}$ remain, specifically denoted as **E2**.

*Case 3*: When $c_{1}<1/2$, strong minority identity of minority A, $s_{1}>1/2$, high status role for language A, the phenomenon of language complete extinction is formed and language minority identity is stronger than language status, only individuals $w_{1}$ remain, specifically denoted as **E3**.

*Case 4*: When $c_{2}<1/2$, strong minority identity of minority B, $s_{1}<1/2$, high status role for language B, the phenomenon of language complete extinction is formed and language minority identity is stronger than language status, only individuals $w_{2}$ remain, specifically denoted as **E4**.

**Figure 2.**
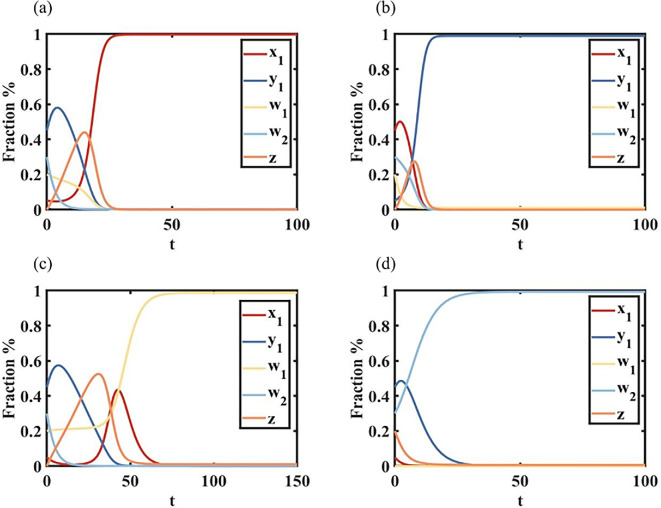
Graph of language complete extinction over time. (a) For $s_{1}=0.7$, $c_{1}=0.8$, $c_{2}=0.8$, we show a situation of only individuals $x_{1}$ exist. (b) For $s_{1}=0.1$, $c_{1}=0.9$, $c_{2}=0.9$, we show a situation of only individuals $y_{1}$ exist. (c) For $s_{1}=0.7$, $c_{1}=0.4$, $c_{2}=0.7$, we show a situation of only individuals $w_{1}$ exist. (d) For $s_{1}=0.2$, $c_{1}=0.7$, $c_{2}=0.4$, we show a situation of only individuals $w_{2}$ exist.

**Figure 3.**
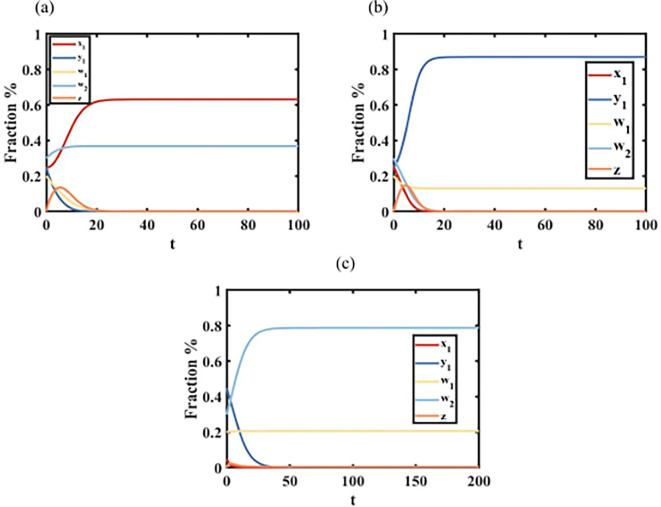
Graph of language segregation over time. (a) For $s_{1}=0.6$, $c_{1}=0.7$, $c_{2}=0.4$, we show a situation of segregation of individuals $x_{1}$ and individuals $w_{2}$. (b) For $s_{1}=0.1$, $c_{1}=0.9$, $c_{2}=0.9$, we show a situation of segregation of individuals $y_{1}$ and individuals $w_{1}$. (c) For $s_{1}=0.2$, $c_{1}=0.5$, $c_{2}=0.4$, we show a situation of segregation of individuals $w_{1}$ and individuals $w_{2}$.

Proposition 1 shows a phenomenon of language complete extinction. Figure 2a shows that when $s_{1}=0.7$, $c_{1}=0.8$, $c_{2}=0.8$, i.e. $s_{1}>1/2$, language A is in a higher status in the area, the role of minority identity of minority A and minority identity of minority B are relatively low, and there is a phenomenon of language extinction, which means of all the languages only language A exists. The initial condition $\left(x_{10},y_{10},w_{10},w_{20}\right)=(0.05,0.45,0.2,0.3)$, due to the language shift to $y_{1}\to z\to x_{1}$, and $w_{1}\to x_{1}$, $w_{2}\to y_{1}\to z\to x_{1}$, the proportion of individuals $x_{1}$ increases in the group. As individuals $y_{1}$ in the group are affected by the status of language A, they are first transferred from individuals $y_{1}$ to bilingual individuals z, so that individuals $w_{1}$ first increase and then are fully extinct over time, and finally only individuals $x_{1}$ remain. Figure 2b shows that similar to figure 3a, when $s_{1}=0.1$, $c_{1}=0.9$, $c_{2}=0.9$, i.e. $s_{1}<1/2$, language B is in a higher status in the area, the roles of minority identity of minority A and minority identity of minority B are relatively low, and there is a phenomenon of language extinction, which means of all the languages only individuals $y_{1}$ exists. Figure 2c shows that when $s_{1}=0.7$, $c_{1}=0.4$, $c_{2}=0.7$, i.e. $c_{1}<1/2$, minority identity of minority A in the area is stronger, the status of language A and the role of minority identity of minority B is relatively low, another phenomenon of language extinction occurs, there are only $w_{1}$ individuals in the group. Initial conditions $\left(x_{10},y_{10},w_{10},w_{20}\right)=(0.05,0.45,0.2,0.3)$, due to the language shift to $y_{1}\to z\to x_{1}$, and $x_{1}\to w_{1}$, $w_{2}\to y_{1}\to z\to x_{1}\to w_{1}$, the proportion of individuals $w_{1}$ increases in the group, whereas the proportions of bilingual individuals z but individuals $x_{1}$first increase and then decrease. Figure 2d shows that when $s_{1}=0.2$, $c_{1}=0.7$, $c_{2}=0.4$, i.e. $c_{2}<1/2$, the minority identity of minority B in the area is stronger, the status of language A and the role of the minority identity of minority B are relatively low, and another phenomenon of language extinction, i.e. there are only $w_{2}$ individuals in the group.

*Proposition 2. Language segregation*

*Case 1*: When $c_{2}<1/2$, $s_{1}>1/2$, strong identity of minority B and language B of status is higher, $c_{1}>1/2$, weak identity of minority A, individuals $w_{2}$ segregate from individuals $x_{1}$, appearance language segregation, specifically denoted as **E5**.

*Case 2*: When $c_{1}<1/2$, $s_{1}<1/2$, strong identity of minority A and language A of status is higher, $c_{2}>1/2$, weak identity of minority B, individuals $w_{1}$ segregate from individuals $y_{1}$, appearance language segregation, specifically denoted as **E6**.

*Case 3*: When $c_{1}<1/2$, $c_{2}<1/2$, strong identity of minority A and strong identity of minority B, $s_{1}<1/2$, the phenomenon of complete coexistence $w_{1}$ and $w_{2}$ is formed.

Proposition 2 shows a phenomenon of language segregation. Figure 3a shows that when $s_{1}=0.6$, $c_{1}=0.7$, $c_{2}=0.4$, minority identity of minority B and language B status interactions and identity of minority B are greater, allowing the formation of individuals $x_{1}$, with the presence of individuals $w_{2}$, and leaving the two individuals in the group with no actual communication, creating a segregated state. Figure 3b shows that when $s_{1}=0.2$, $c_{1}=0.4$, $c_{2}=0.7$, minority identity of language A and language A status interactions and the minority identity of language A are greater, creating a situation where individuals $y_{1}$ exists in isolation from individuals $w_{1}$. For example, in a county in Pu'er City, Yunnan Province, there are minority Wa, minority Lahu and other ethnic minority groups. For the minority Wa individual $w_{2}$ has a strong sense of minority identity, which makes him or her speak only the minority Wa language, whereas the minority Lahu individual holds an open attitude towards foreign cultures or languages, which forms the minority Wa-only individual $w_{2}$ and the minority Lahu-speaking individual $x_{1}$. The minority Wa individual $w_{2}$ and Lahu-speaking individual $x_{1}$ have been formed in the same county. Figure 3c shows that when $s_{1}=0.2$, $c_{1}=0.4$, $c_{2}=0.4$, identity of minority A is high and identity of minority B is high, there are $w_{1}$ individuals and $w_{2}$ individuals in the group, who do not interfere with each other and maintain linguistic isolation. We got through the research that there are Dai and Brown ethnic groups surviving in Jingmai Mountain in Pu'er City, and they have a strong ethnic identity in terms of their culture and language, so that they are distributed at the foot and top of the same mountain slope, and the minority groups have a clear demarcation line.

*Proposition 3. Language coexistence*

*Case 7*: When $s_{1}<1/2$, $c_{1}=1/2$,$\;c_{2}=1/2$, as the role of minorities identity B increases, it allows the formation of the phenomenon of coexistence of individuals $y_{1}$ and $w_{1}$, $w_{2}$.

*Case 8*: When $s_{1}>1/2$, $c_{1}=1/2$, $c_{2}=1/2$, as the role of minorities identity A increases, it allows the formation of the phenomenon of coexistence of individuals $x_{1}$ and $w_{1}$, $w_{2}$.

**Figure 4.**
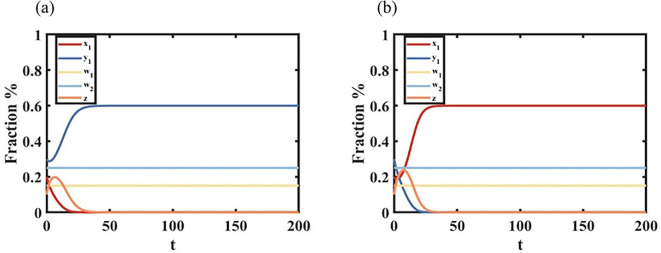
Graph of language coexistence status over time. (a) For $s_{1}=0.6$, $c_{1}=0.7$, $c_{2}=0.4$, we show a situation of coexistence of individuals $y_{1},w_{1}$ and $w_{2}$. (b) For $s_{1}=0.1$, $c_{1}=0.9$, $c_{2}=0.9$, we show a situation of coexistence of individuals $x_{1},w_{1}$ and $w_{2}$.

Proposition 3 shows a phenomenon of language coexistence. Figure 4a shows that when $s_{1}=0.4$, $c_{1}=0.5$, $c_{2}=0.5$, language B of status interacts with identity of minority B, individuals $y_{1}$ and individuals $w_{2}$, as well as individuals $w_{1}$, appear in the group. Figure 4b shows that when $s_{1}=0.6$, $c_{1}=0.5$, $c_{2}=0.5$, language A of status interacts with identity of minority A individuals $x_{1}$ and individuals $w_{1}$, as well as individuals $w_{2}$, appear in the group.

The above balance illustrates the diverse linguistic phenomena and how language diffusion is influenced by the interplay of language status and language minority identity. Language status motivates individuals to adopt a dominant or more favourable language for social and economic reasons. In contrast, minority identity represents a desire to maintain one’s heritage language as a marker of cultural or group identity. In minority groups, minority identity is important, even more so than language status. The balance between these interactions leads to different outcomes, including language complete extinction, language segregation and language coexistence.

## 4. The influence of self-minority identity transmission in complex network

So far, we have neglected the effect of fluctuations because the population is considered large and our deterministic approximation results are valid in the thermodynamic limit of infinite systems. To build a more realistic underlying contact network, we consider computations and simulations in complex networks. Specifically, we select two types of classical network models. For homogeneous networks, we employ the ER (Erdős-Rényi) random network [37], which is characterized by a uniform probability of edge connections between nodes, with an average degree set to $\langle k\rangle =20$. This structure provides a simplified reference framework, facilitating the analysis of the fundamental features of system behaviour. Considering that real-world social networks are generally heterogeneous and exhibit scale-free properties, we adopt the BA (Barabási-Albert) network [38] for modelling. The BA network captures the non-uniform connectivity among nodes, particularly highlighting the significant influence of a few core nodes on the propagation process. The other parameter settings remain consistent with those of the homogeneous network to enable comparative analysis. Through simulations on both homogeneous and heterogeneous networks, we aim to reveal the impact of different network structures on the dynamics of language diffusion.

To more accurately parse the communication mechanisms between different language groups, we make more precise assumptions about local communication events and construct a network-based model of language diffusion. It is assumed that there are N individuals in the system, and the individuals are connected to each other through edges in the network, and the influence of external immigrant individuals is not considered. Individuals in the system are divided into two groups: group W, which represents individuals who only use their own minority language and oppose foreign minority cultures, where $W=w_{1}+w_{2}$; and group X, which represents individuals who support foreign minority cultures, and who not only use their own minority language but also have the ability to learn other minority languages, where $X=x_{1}+y_{1}$.

**Figure 5.**
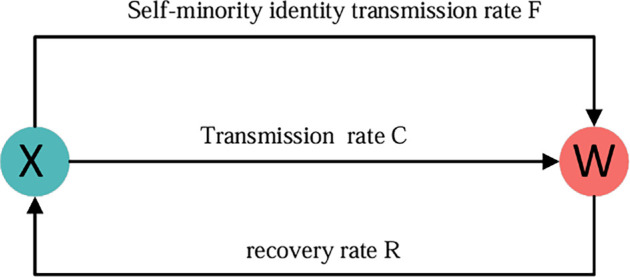
The schematic diagram of language diffusion with self-minority identity transmission F. This society consists of many speakers who speak with other individuals. The speakers are categorized into two states, minority individuals W can only speak their own minority language and individuals X can speak their own language and learn other minority languages.

In the model, language diffusion depends on the connecting edges between individuals in the network. When there is a connecting edge between two individuals, minority identity may be transmitted when individual X comes into contact with individual W. The probability of successful transmission is C. In addition, individual X, as a member of a minority, can also enhance its minority identity through self-learning to become individual W. The process of self-learning minority identity is influenced by the minority identity level of an individual’s neighbours, with the self-learning minority identity transmission rate described as $F\cdot X_{i}\cdot W\left(X_{i}\right)$, where $W(X_{i})$ denotes the proportion of opponents among the neighbours of node i at time t. Meanwhile, individuals in group *W* may transition to group X. This process is characterized by a recovery rate R, reflecting the interaction intensity between individuals and their neighbours who support foreign cultures. Under this mechanism, we utilize the network structure to portray the micro communication process between different language groups, revealing the dynamic evolution pattern of minority identity and language diffusion, as shown in figure 5. This hypothesis and model closely resemble the phenomenon of language communication with minority identity in real social networks, which provides an important theoretical basis for an in-depth understanding of the diffusion path and evolution mechanism of minority languages.

Let $X_{i}\left(t\right),W_{i}\left(t\right)$ be the relative density of supporters and opponents of foreign minority culture with degree k at time t, which satisfy 0 $\leq X_{i}\left(t\right),W_{i}\left(t\right)<1$ and $X_{i}\left(t\right)+W_{i}\left(t\right)=1$. The initial conditions for minority identity transmission are $X_{i}\left(0\right),W_{i}\left(0\right)$. Under the assumption that the networks are uncorrelated, the conditional probability is $p\left(\left.k^{`}\right|k\right)=k^{`}p(k^{`})/\langle k\rangle$. Let Θ(t) be the probability that any given link points to an opponent at time t. Then Θ(t) can be written as


(4.1) $$\Theta \left(t\right)=\frac{1}{\left\langle k\right\rangle }\sum_{k^{\prime }}k^{\prime }p\left(k^{\prime }\right)W_{k^{\prime }}\left(t\right),\;\;\;\;\;\;\;\;\;\;\;\;\;\;\;\;\;\;\;\;\;\;\;\;\;\;\;\;\;\;\;\;\;\;\;\;\;\;\;\;\;\;\;\;\;\;\;\;\;\;\;$$

where $\langle k\rangle =\sum_{k}kp(k)$ is the average degree of the network.

Then, the self-minority identity transmission process can be denoted by $F\cdot X_{i}\cdot \Theta$. Therefore, the mean field equations of the model with self-minority identity transmission in BA network can be written as


(4.2) $$\left\{ \begin{array}{l} \frac{dX_{k}\left(t\right)}{dt}=-CX_{k}\left(t\right)k\Theta -FX_{k}\left(t\right)\Theta +RW_{k}\left(t\right),\\ \frac{dW_{k}\left(t\right)}{dt}=CX_{k}\left(t\right)k\Theta +FX_{k}\left(t\right)\Theta -RW_{k}\left(t\right). \end{array}\right.\;\;\;\;\;\;\;\;\;\;\;\;\;\;\;\;\;\;\;\;\;\;\;\;$$

For simplicity, we set a unitary forgetting rate R=1 and substitute $X_{k}\left(t\right)$ by $1-W_{k}\left(t\right)$. Equation (4.2) can be rewritten as


(4.3) $$\frac{dW_{k}\left(t\right)}{dt}=C\left(1-W_{k}\left(t\right)\right)k\Theta +FX_{k}\left(t\right)\Theta -W_{k}\left(t\right).\;\;\;\;\;\;\;\;\;\;\;\;\;\;\;\;$$

Let $dW_{k}(t)/dt=0$, we obtain


(4.4) $$W_{k}\left(t\right)=\frac{Ck\Theta +F\Theta }{1+Ck\Theta +F\Theta }.\;\;\;\;\;\;\;\;\;\;\;\;\;\;\;\;\;\;\;\;\;\;\;\;\;\;\;\;\;\;\;\;\;\;\;\;\;\;\;\;\;\;\;\;\;\;\;\;\;\;\;\;\;\;\;\;\;\;\;\;\;\;$$

In the steady state, $W_{k}\left(t\right)$ is a function of Θ. Moreover, we can derive the self-consistency equation of Θ by substituting equation (4.4) into equation (4.1):


(4.5) $$\Theta =\frac{1}{\left\langle k\right\rangle }\sum_{k^{\prime }}k^{\prime }p\left(k^{\prime }\right)\frac{Ck^{\prime }\Theta +F\Theta }{1+Ck^{\prime }\Theta +F\Theta }.\;\;\;\;\;\;\;\;\;\;\;\;\;\;\;\;\;\;\;\;\;\;\;\;\;\;\;\;\;\;\;\;\;\;\;\;\;\;\;\;\;\;\;\;$$

Obviously, Θ=0 is a solution of equation (4.5). Then, we derive the conditions under which a non-trivial solution to equation (4.5) exists. We define a function $f\left(\Theta \right)=\Theta -\frac{1}{\left\langle k\right\rangle }\sum_{k^{\prime }}k^{\prime }p\left(k^{\prime }\right)\frac{Ck^{\prime }\Theta +F\Theta }{1+Ck^{\prime }\Theta +F\Theta }$ and its first derivative and second derivative


(4.6) $$\frac{df\left(\Theta \right)}{d\Theta }=1-\frac{1}{\left\langle k\right\rangle }\sum_{k^{\prime }}k^{\prime }p\left(k^{\prime }\right)\frac{Ck^{\prime }+F}{{\left(1+Ck^{\prime }\Theta +F\Theta \right)}^{2}}.\;\;\;\;\;\;\;\;\;\;\;\;\;\;\;\;\;\;\;\;\;\;$$

(4.7) $$\frac{d^{2}f\left(\Theta \right)}{d\Theta }=\frac{2}{\left\langle k\right\rangle }\sum_{k^{\prime }}k^{\prime }p\left(k^{\prime }\right)\frac{{\left(Ck^{\prime }+F\right)}^{2}}{{\left(1+Ck^{\prime }\Theta +F\Theta \right)}^{3}}.\;\;\;\;\;\;\;\;\;\;\;\;\;\;\;\;\;\;\;\;\;\;\;\;\;$$

It is easy to see that $d^{2}f\left(\Theta \right)/d\Theta >0$. Then $f\left(\Theta \right)$ is a convex function with satisfying $f\left(0\right)=0$ and f(1)>0. Therefore, $\frac{df(\Theta )}{d\Theta }{|}_{\Theta =0}=1-\left(F+C\frac{\left\langle k^{2}\right\rangle }{\left\langle k\right\rangle }\right)<0$ must be satisfied. The self-minority identity transmission threshold is $C=(1-F)\frac{\langle k\rangle }{\langle k^{2}\rangle }$. Then in the ER network, the self-minority identity transmission threshold is $C=(1-F)\frac{1}{\langle k\rangle }$.

**Figure 6.**
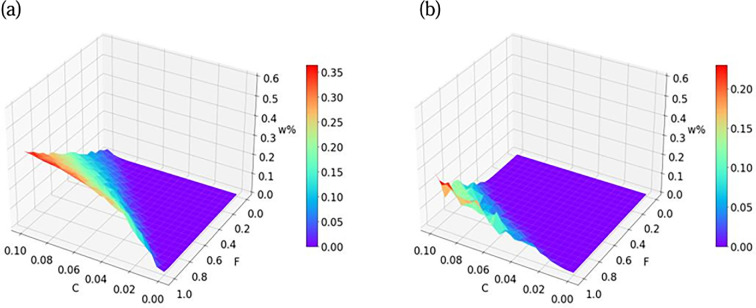
The final density of individuals W as a function of transmission rate C and self-minority identity transmission rate F. (a) BA network. (b) ER network. Network of $N=10^{3}$ nodes and for $t=10^{2}$ Monte Carlo steps. The colour values are calculated as an average over 20 realizations.

We conduct simulations on the ER random network and the BA network, where the number of nodes is $N=10^{3}$. The BA network has a degree distribution satisfying $p(k)\sim k^{-r}$ with the degree exponent r=3 and $\langle k\rangle \approx 6,\langle k^{2}\rangle \approx 86$. The ER random network satisfies $\langle k\rangle \approx 6$. Figure 6 shows the density of individuals *W* under different rates of self-minority identity transmission F. Under a certain transmission rate C, the density of individuals W in the group increases that increasing self-minority transmission rate, and the self-minority identity transmission rate has an obvious positive effect on individuals W, both in the ER and the BA networks, especially in the BA network. Therefore, the BA network is more favourable for minority language diffusion. It should start with basic self-awareness enhancement, and provide and encourage more learning of minority knowledge and minority culture, which is more conducive to the preservation of the minority languages. To protect minority languages, we can spread the individuals with greater connectivity, which makes the diffusion of minority languages more rapid and is conducive to the preservation of minority languages.

**Figure 7.**
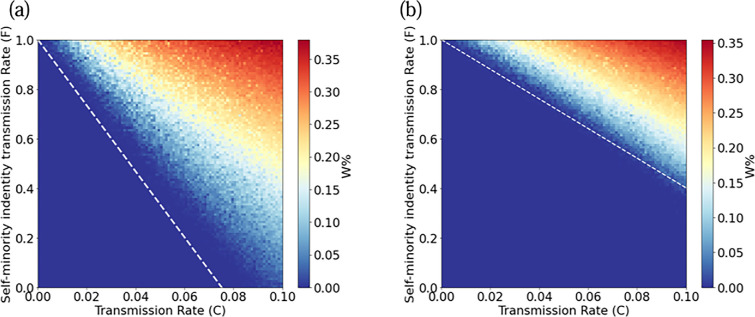
Phase diagram of individuals W for R=1. With white, discontinuous lines we plot the boundaries between different phases determined. The lines are plotted over the results of a simulation of the model in (a) BA network (b) ER network of $N=10^{3}$ nodes and for $t=10^{2}$ Monte Carlo steps. The colour values are calculated as an average over 10 realizations.

To better verify the consistency of the model and theory, Monte Carlo simulation is used. We plot the phase diagram of the density of individuals W in complex networks. Transmission rate C and self-minority identity transmission rate F as a function of individuals W. We find that as the value of F increases, the number of individuals W increases. Meanwhile, the white dashed line represents the theoretical threshold. The theoretical thresholds of the simulations agree well with the numerical simulations (figure 7a,b). It is obvious that the BA network is easier to minority languages, which is realized by the characteristics of BA network. For minority language preservation institutions and policies, highly connected nodes or influencers in the network should be encouraged to speak minority languages to increase minority language diffusion. Increasing self-minority identity transmission rate F can increase individuals W and be more conducive to minority preservation.

**Figure 8.**
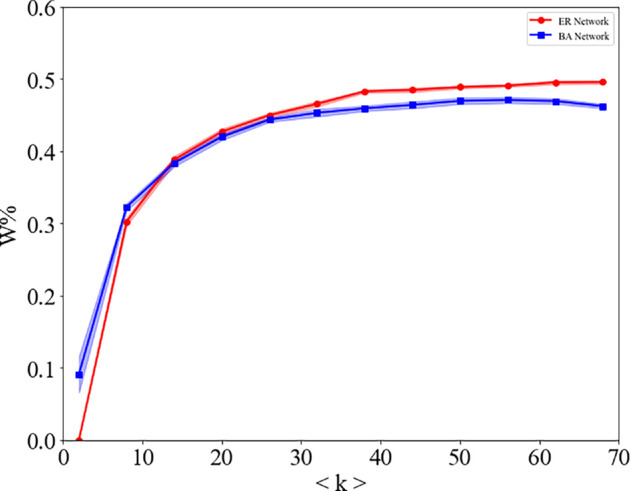
The final proportion of individuals W as function of the average degree <k> of the network. Red circles represent ER networks and blue squares represent BA networks of $N=10^{3}$ nodes with average degree <k> ranging from 2 to 68 and for $t=10^{2}$ Monte Carlo steps. The colour values are calculated as an average over 20 simulations and the shaded regions represent the 90% reference range.

Hitherto, we have shown that self-minority identity transmission plays an important role in minority language diffusion. Further research on the topology of the network is needed to investigate this positive role. In figure 8, we show the final proportion of individuals W as function of the average degree $\langle k\rangle$ of the network. Red circles represent ER networks and blue squares represent BA networks of $N=10^{3}$ nodes. It is known that networks’ topological features affect and drive spreading patterns taking place on top of them [39]. Therefore, we take networks with different connectivity patterns as the objects of study and investigate the effect of network topology on the whole dynamic process. Specifically, we use the BA network and the classical ER network. Although there are differences in the results obtained due to the different structures of homogeneous and heterogeneous networks, they obtained consistent conclusions: increasing the average degree of network, whether they are heterogeneous or homogeneous, can promote minority language to some extent, thereby promoting minority language diffusion.

## 5. Validation of real minority social networks

To further support the theoretical results obtained in the previous section, we analyse language diffusion with minority identity on real-world networks. Specifically, we investigate the identity of minority languages in Ximeng County, Pu'er City, Yunnan Province, to obtain the phenomenon of language segregation. We sampled 11 groups according to the stratified sampling method from 1748 people in Masan Village, Ximeng County, in which 37 minority Lahu individuals and 140 minority Wa individuals were proportionally sampled, in which minority identity was scored according to the scale, with a score of 10 out of 10, and individuals of Lahu with minority identity of 6 or more accounted for 7/37, and Wa individuals' minority identity is 130/140. According to the survey results, minority Wa individuals speak only minority Wa language, while minority Lahu individuals are open to the minority Wa language, thus forming the phenomenon of language segregation between minority Wa individuals with a strong minority identity and minority Lahu individuals with a sense of openness to the outside world. Within minority groups, there are also individuals who support foreign languages and those who oppose them.

**Figure 9.**
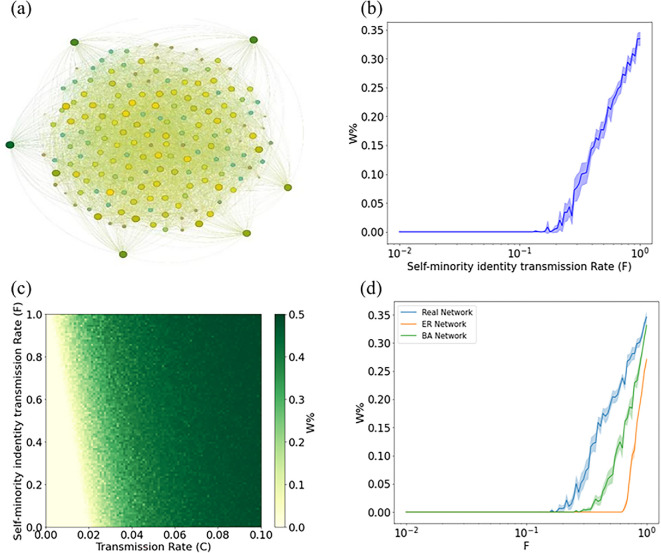
Results from numerical simulations of minority language transmission in a real social network. (a) Minority individual social networks. (b) Individuals **W** supporting own minority language as function of *F* for C=0.015 averaged over 30 simulations and the shaded regions represent the 90% reference range. (c) The simulated phase diagram of individualW supporting own minority language in network a for R=1 averaged over 30 simulations. (d) Comparison of simulated phase diagrams of individualsW supporting their own language in the three networks.

To study the influence of minority identity on the diffusion of minority languages, we investigated the relationships obtained from real social networks to simulate the language diffusion patterns of minority languages in real social networks. We construct a social network in which the nodes are minority individuals who are socially connected to different minority groups. In particular, connections represent social relationships where they are friends or family. Figure 9a shows the form of social network specific connections. Our calculations show that self-minority identity transmission *F* contributes to the diffusion of minority languages, and there is a significant shift in the threshold for minority language diffusion (figure 9b). In figure 9c, we show the effects of C and F on the final proportion of W. Self-minority identity transmission on real social networks has a positive effect on ethnolinguistic diffusion, and self-minority identity transmission can contribute to the preservation of minority languages by increasing individual minority identity. In this network, the theoretical conclusions are not easy to compute as the data from real social networks extend the modelling framework, then the assumption of uncorrelated network structure in the computation is no longer valid. However, we do observe a similar minority language diffusion phenomenon in real social networks, where self-minority identity transmission F does have a key transition point.

To further validate the practical applicability of the model, we compared the theoretical simulation results with the transmission process in real minority social networks, as shown in figure 9d. The comparative analysis reveals that there are similarities and differences between the language diffusion thresholds in real minority social networks and the simulated ones. *Similarities*: in all network structures (BA network, ER network and real minority social network), the proportion of individuals W increases significantly with increasing self-minority identity transmission rate F, indicating that the self-learning minority identity mechanism plays an important role in facilitating the language transmission process. *Differences*: the transmission thresholds in the real minority social networks were greater than those in the ER and BA networks, suggesting that local structural features in the real minority social networks have an impact on the language transmission paths, thus increasing the critical conditions for language transmission. This difference emphasizes the complex influence of network topology on the language transmission process.

## 6. Conclusion

In this work, we explore the role of minority identity in interethnic competition in the same area. We address the influence of minority identity by proposing a model that takes into account minority identity preferences and categorizes minority individuals A into those who support foreign language individuals and those who oppose foreign languages. Assuming first that the model is a homogeneous mixed mean field, depending on the degree of minority identity and the status of the language, we obtain different forms of minority languages, namely, language complete extinction, language segregation and language coexistence. Language extinction, i.e. there are only individuals $x_{1}(x_{2}$) or only individuals $w_{1}(w_{2}$) who support only their own minority language $A\left(B\right)$ in the same area. For example, the Dulong people, who live in China’s Yunnan Province, have slowly lost their language over time. Language segregation is the result of competition between minority identity and language status, and relatively strong minority ideology that supports only individuals in their own language may also survive in equilibrium, leading to the segregation of individuals $w_{1}$ and individuals $y_{1}$, individuals $w_{2}$ and individuals $x_{1}$. For example, in the Greater Miami area of each state of Florida, from 1960 to the early 1990s, language competition occurred in the area due to Cuban immigration, which led to language segregation by minority identity and local language competition [40]. Language coexistence, which indicates the presence of bilingualism in the system, greatly protects language diversity and allows for language coexistence. Linguistic phenomena can be observed in multilingual societies around the world, where historical, social and political contexts shape the linguistic landscape in complex ways. Our research provides valuable insights for understanding and predicting ethnolinguistic change and a framework for decision making in language planning, education, and cultural preservation.

Second, we added language diffusion with self-minority identity, considered the diffusion process in homogeneous and heterogeneous networks, derived diffusion thresholds of the self-minority identity diffusion rate *F* and verified the theoretical results by numerical experiments. Numerical results show that self-minority identity transmission rate positively contributes to minority language diffusion in both BA networks and ER networks. In addition, the BA network is more efficient in spreading. Overall, ethnolinguistic diffusion is affected not only by the rate of self-ethnic awareness diffusion but also by the structure of the network. Improving the average degree of network, whether heterogeneous or homogeneous, can promote the development of minority languages to some extent, and thus promote the diffusion of minority languages.

Finally, we validate the language diffusion model with self-minority identity by constructing real social networks in the minority Wa region of Yunnan. The results of the modelling and analytical validation presented in this article emphasize the importance of considering the complex structure and self-learning in social systems when it comes to the social communication process. The results of the study show that the process of minority language diffusion exhibits a phase transition, and the critical threshold depends on the structure of the network and the diffusion of self-minority identity. The flexibility of the framework allows for further research into different types of diffusion behaviours that may be more complex than the models used here. For example, to enable minority language diffusion, the process of interactions between individuals can be more complex, such as incorporating age-specific patterns of exposure and education, the influence of different geographic contexts and data-driven human mobility. These features have the potential to provide not only unexpected theoretical results but also actionable insights important for understanding and controlling socio-ethnic linguistic communication processes. A notable strength of this study is that we theoretically analyse the proposed model and validate it using real-world data, which are often difficult to obtain. However, the data are currently limited to a specific region, which may restrict the broader applicability of our results. In future research, we aim to collect and analyse data from additional regions to enhance the generalizability of the findings and improve their practical relevance. Expanding the scope of our data will allow us to more accurately capture the dynamics of language diffusion across diverse regions, thereby increasing the robustness and impact of our study.

## Data Availability

Data and relevant code for this research work are stored in GitHub [https://github.com/elegent0325/minority-language.git] and have been archived within the Zenodo repository [41].

## References

[1] Rudi K. 1994 On language change: the invisible hand in language. London, UK: Routledge.

[2] Currie TE, Mace R. 2009 Political complexity predicts the spread of ethnolinguistic groups. Proc. Natl Acad. Sci. USA **106**, 7339–7344. (10.1073/pnas.0804698106) 19380740 PMC2670878

[3] Bentz C, Dediu D, Verkerk A, Jäger G. 2018 The evolution of language families is shaped by the environment beyond neutral drift. Nat. Hum. Behav. **2**, 816–821. (10.1038/s41562-018-0457-6) 31558817

[4] Sutherland WJ. 2003 Parallel extinction risk and global distribution of languages and species. Nature **423**, 276–279. (10.1038/nature01607) 12748639

[5] Baggs I, Freedman HI. 1990 A mathematical model for the dynamics of interactions between a unilingual and a bilingual population: persistence versus extinction. J. Math. Sociol. **16**, 51–75. (10.1080/0022250x.1990.9990078)

[6] Abrams DM, Strogatz SH. 2003 Linguistics: modelling the dynamics of language death. Nature **424**, 900–900. (10.1038/424900a) 12931177

[7] Mira J, Paredes Á. 2005 Interlinguistic similarity and language death dynamics. Europhys. Lett. **69**, 1031–1034. (10.1209/epl/i2004-10438-4)

[8] Minett JW, Wang WSY. 2008 Modelling endangered languages: the effects of bilingualism and social structure. Lingua **118**, 19–45. (10.1016/j.lingua.2007.04.001)

[9] Gao Y, Liu W. 2023 Measures to sustain endangered languages: a bilingual competition model with sliding mode control. PLoS One **18**, e0287850. (10.1371/journal.pone.0287850) 37384628 PMC10309631

[10] Marco P, Els H. 2009 Influence of geography on language competition. Phys. A **388**, 174–186. (10.1016/j.physa.2008.09.034)

[11] Mussa Juane M, Seoane LF, Muñuzuri AP, Mira J. 2019 Urbanity and the dynamics of language shift in Galicia. Nat. Commun. **10**, 1680. (10.1038/s41467-019-09688-8) 30976005 PMC6459816

[12] Brown D, Wrathmall S. 2023 Geographical modelling of language decline. R. Soc. Open Sci. **10**, 221045. (10.1098/rsos.221045) 37234501 PMC10206464

[13] Prochazka K, Vogl G. 2017 Quantifying the driving factors for language shift in a bilingual region. Proc. Natl Acad. Sci. USA **114**, 4365–4369. (10.1073/pnas.1617252114) 28298530 PMC5410847

[14] Zhang MH, Gong T. 2013 Principles of parametric estimation in modeling language competition. Proc. Natl Acad. Sci. USA **110**, 9698–9703. (10.1073/pnas.1303108110) 23716678 PMC3683775

[15] Hua X, Greenhill SJ, Cardillo M, Schneemann H, Bromham L. 2019 The ecological drivers of variation in global language diversity. Nat. Commun. **10**, 1–10. (10.1038/s41467-019-09842-2) 31053716 PMC6499821

[16] Laila MA. 2023 Language attitudes in fast-growing societies: new insights in the dynamism dimension. Humanit. Soc. Sci. Commun. **10**, 1–14. (10.1057/s41599-023-01988-1)

[17] Amin A. 2020 Attitude towards language in sociolinguistics settings: a brief overview. REiLA **2**, 27–30. (10.31849/reila.v2i1.3758)

[18] Cargile AC, Giles H. 1994 Language attitudes as a social process: a conceptual model and new directions. Lang. Commun. **14**, 211–236. (10.1016/0271-5309(94)90001-9)

[19] El‐Dash LG, Busnardo J. 2001 Brazilian attitudes toward English: dimensions of status and solidarity. Int. J. Appl. Linguist. **11**, 57–74. (10.1111/1473-4192.00004)

[20] Garrett P. 2010 Attitudes to language. New York, NY, USA: Cambridge University Press.

[21] Garrett P. 2007 Language attitudes. Chapter 14. In The Routledge companion to sociolinguistics (eds L Carmen, M Louise), pp. 133–139. London, UK: Routledge.

[22] Sallabank J. 2013 Attitudes to endangered languages: identities and policies. New York, NY, USA: Cambridge University Press.

[23] Barreira da Silva Rocha A. 2018 Social outcomes due to the interplay between language competition and ideology struggle. Physica. A **492**, 1340–1351. (10.1016/j.physa.2017.11.061)

[24] Fibich G. 2016 Bass-SIR model for diffusion of new products in social networks. Phys. Rev. E **94**, 032305. (10.1103/physreve.94.032305) 27739743

[25] Harko T, Lobo SN, Mak MK. 2014 Exact analytical solutions of the susceptible-infected-recovered (SIR) epidemic model and of the SIR model with equal death and birth rates. Appl. Math. Comput. **236**, 184–194. (10.1016/j.amc.2014.03.030)

[26] Zhang Z, Mei XH, Jiang H, Luo X, Xia Y. 2023 Dynamical analysis of hyper-SIR rumor spreading model. Appl. Math. Comput. **446**, 127887. (10.1016/j.amc.2023.127887)

[27] Jeffs RA, Hayward J, Roach PA, Wyburn J. 2016 Activist model of political party growth. Physica. A **442**, 359–372. (10.1016/j.physa.2015.09.002)

[28] Xia CY, Wang ZS, Zheng CY, Guo QT, Shi YT, Dehmer M, Chen ZQ. 2019 A new coupled disease-awareness spreading model with mass media on multiplex networks. Inf. Sci. **471**, 185–200. (10.1016/j.ins.2018.08.050)

[29] Qiu L, Jia W, Niu W, Zhang M, Liu S. 2021 SIR-IM: SIR rumor spreading model with influence mechanism in social networks. Soft Comput. **25**, 13949–13958. (10.1007/s00500-020-04915-7)

[30] Albury N. 2020 Language attitudes and ideologies on linguistic diversity. Chapter 18. In Handbook of home language maintenance and development (eds A Schalley, S Eisenchla), pp. 357–376. Boston, MA: De Gruyter Mouton. (10.1515/9781501510175-018)

[31] Garrett P. 2001 Language attitudes and sociolinguistics. J. Socioling. **5**, 626–631. (10.1111/1467-9481.00171)

[32] Milroy J. 2007 The ideology of the standard language. Chapter 16. In The Routledge companion to sociolinguistics (eds L Carmen, M Louise, S Peter), pp. 133–139. London, UK: Routledge.

[33] Iñiguez G, Heydari S, Kertész J, Saramäki J. 2023 Universal patterns in egocentric communication networks. Nat. Commun. **14**, 5217. (10.1038/s41467-023-40888-5) 37633934 PMC10460427

[34] Wang HY, Wang J, Ding LT, Wei W. 2017 Knowledge transmission model with consideration of self-learning mechanism in complex networks. Appl. Math. Comput. **304**, 83–92. (10.1016/j.amc.2017.01.020)

[35] Yan Z, Gao J, Wang S, Lan Y, Xiao J. 2023 Investigation on the influence of heterogeneous synergy in contagion processes on complex networks. Chaos **33**, 073147. (10.1063/5.0152516) 37477606

[36] Heinsalu E, Patriarca M, Léonard JL. 2014 The role of bilinguals in language competition. Adv. Complex Syst. **17**, 1450003. (10.1142/s0219525914500039)

[37] Erdos P, Renyi A. 1960 On the evolution of random graphs. Inst. Hung. Acad. Sci. **5**, 17–61. (10.1515/9781400841356.38)

[38] Barabási AL, Albert R. 1999 Emergence of scaling in random networks. Science **286**, 509–512. (10.1126/science.286.5439.509) 10521342

[39] Garcia MC. 2004 Exiles, immigrants, and transnationals: the Cuban communities of the United States. In The Columbia history of Latinos in the United States since 1960 (ed. DG Gutiérrez), pp. 146–186. New York, NY: Columbia University Press. (10.1017/CBO9780511791383)

[40] Barrat A, Barthelemy M, Vespignani A. 2008 Dynamical processes on complex networks. Cambridge, UK: Cambridge University Press. (10.1017/CBO9780511791383)

[41] 2025 elegent0325/minority-language: minority identity data (v1.0.0). Zenodo*.* (10.5281/zenodo.14989039)

